# Air pollution mitigation can reduce the brightness of the night sky in and near cities

**DOI:** 10.1038/s41598-021-94241-1

**Published:** 2021-07-16

**Authors:** Miroslav Kocifaj, John C. Barentine

**Affiliations:** 1 ICA, Slovak Academy of Sciences, Dúbravská Road 9, 845 03 Bratislava, Slovakia; 2 Faculty of Mathematics, Physics, and Informatics, Comenius University, Mlynská Dolina, 842 48 Bratislava, Slovakia; 3 International Dark-Sky Association, 3223 N. First Avenue, Tucson, AZ 85719 USA; 4 Consortium for Dark Sky Studies, University of Utah, 375 S 1530 E, RM 235 ARCH, Salt Lake City, UT 84112-0730 USA

**Keywords:** Atmospheric optics, Astronomy and astrophysics, Environmental impact

## Abstract

Light pollution is a novel environmental problem whose extent and severity are rapidly increasing. Among other concerns, it threatens global biodiversity, nocturnal animal migration, and the integrity of the ground-based astronomy research enterprise. The most familiar manifestation of light pollution is skyglow, the result of the interplay of outdoor artificial light at night (ALAN) and atmospheric scattering that obscures views of naturally dark night skies. Interventions to reduce night sky brightness (NSB) involving the adoption of modern lighting technologies are expected to yield the greatest positive environmental consequences, but other aspects of the problem have not been fully explored as bases for public policies aimed at reducing light pollution. Here we show that reducing air pollution, specifically aerosols, decreases NSB by tens of percent at relatively small distances from light sources. Cleaner city air lowers aerosol optical depth and darkens night skies, particularly in directions toward light sources, due to relatively short path lengths traversed by photons from source to observer. A field experiment demonstrating the expected changes when transitioning from conditions of elevated turbidity to cleaner air validated our hypothesis. Our results suggest new policy actions to augment and enhance existing light pollution reduction techniques targeting lighting technology and design.

## Introduction

The usual approach to reducing skyglow, both over cities as well as seen from distant sites such as protected landscapes and astronomical observatories, is focused on making changes to outdoor lighting; in other words, if the source of the problem is addressed, we expect that symptoms of the problem such as skyglow will respond proportionately^1–7^. We recently confirmed this causality in two lighting experiments conducted in the city of Tucson, U.S. When Tucson modernized its municipally owned street lighting system in 2016–2017, it reduced total system light emissions by 63%. As a consequence of this change, the upward-directed optical radiance of Tucson detected from Earth orbit decreased by approximately 7% and the zenith NSB at distant sites (~ 50 km from the city center) declined by an average of about 15%^7^. More recently, we arranged a test with the Tucson municipal government involving its actively controlled white light-emitting diode (LED) street lights that were installed during the modernization project. On nights when most city street lights were simultaneously dimmed by a factor of 2/3, we detected location-dependent changes in the zenith NSB due to the light dimming of − 6% to 0% over radial city-center distances ranging from 5 to 50 km^8^.

However, reducing skyglow over cities through lighting interventions has limited utility for two reasons. One is that modernization of lighting is seen as a once-per-generation opportunity, and once installed, solid-state lighting (SSL) products are expected to operate reliably in field conditions for 20–30 years. Another reason is that world cities continue to grow as the global population urbanizes, and that growth is happening most quickly in developing economies. Given recent history in other aspects of technology, it is likely that cities in those places will “leap over” earlier outdoor lighting technologies and proceed directly to SSL. In this sense, SSL represents both challenge and promise in terms of its impact on skyglow. World demand for ALAN is increasing, and that demand appears to be fueled by the availability of cheap, reliable and energy-efficient SSL^2^; however, the proliferation of blue-rich white light sources such as white LED technology threatens to vastly increase skyglow^1,9^. Therefore, in addition to making changes to sources of light, we must consider other points of leverage on the problem in order to manage the growth of skyglow globally.

We considered aspects of the problem of skyglow formation other than light source characteristics because skyglow is the combination of light sources on the ground plus the light-processing effect of the atmosphere^10^. Although the physics of skyglow formation is well understood, the application of that knowledge as an intervention to reduce its prevalence does not seem to have been suggested in the literature. We hypothesized that persistent declines in atmospheric aerosols resulting from successful iniatives to reduce air pollution would also reduce NSB if all other influences were held fixed. Cleaner air not only has obvious public health benefits^11^, but it could further reduce diffuse artificial light in the night sky and improve astronomical viewing after the utility of other methods such as lighting changes has been exhausted.

The lives of photons emitted by artificial ground-based sources can be manifold due to highly variable atmospheric conditions. Aspects of the light-emitting environment such as the number and spatial distribution of light sources are relatively stable in time, but the type and variability of atmospheric aerosols both show large regional diversity on relatively short time scales. Depending on the concentrations, nature, and sizes of aerosol particles, as well as their transport from air pollution sources, the mean free path of photons can change, potentially shortening the average distance between consecutive scattering events. The more aerosol particles contained in the atmosphere, the more photons tend to scatter downwards, which in turn causes NSB increases as seen from the ground. This effect is strongest near the light sources^9^. At the same time, the concentration of aerosols in the air above cities is in many cases greater than ever. Highly populated regions of the world increasingly confront the problem of rising air pollution. The situation is especially acute in China, where atmospheric aerosol loading has increased by a factor of 10 or more compared to what has been identified in Europe^12,13^.

## Results and discussion

**Figure 1:**
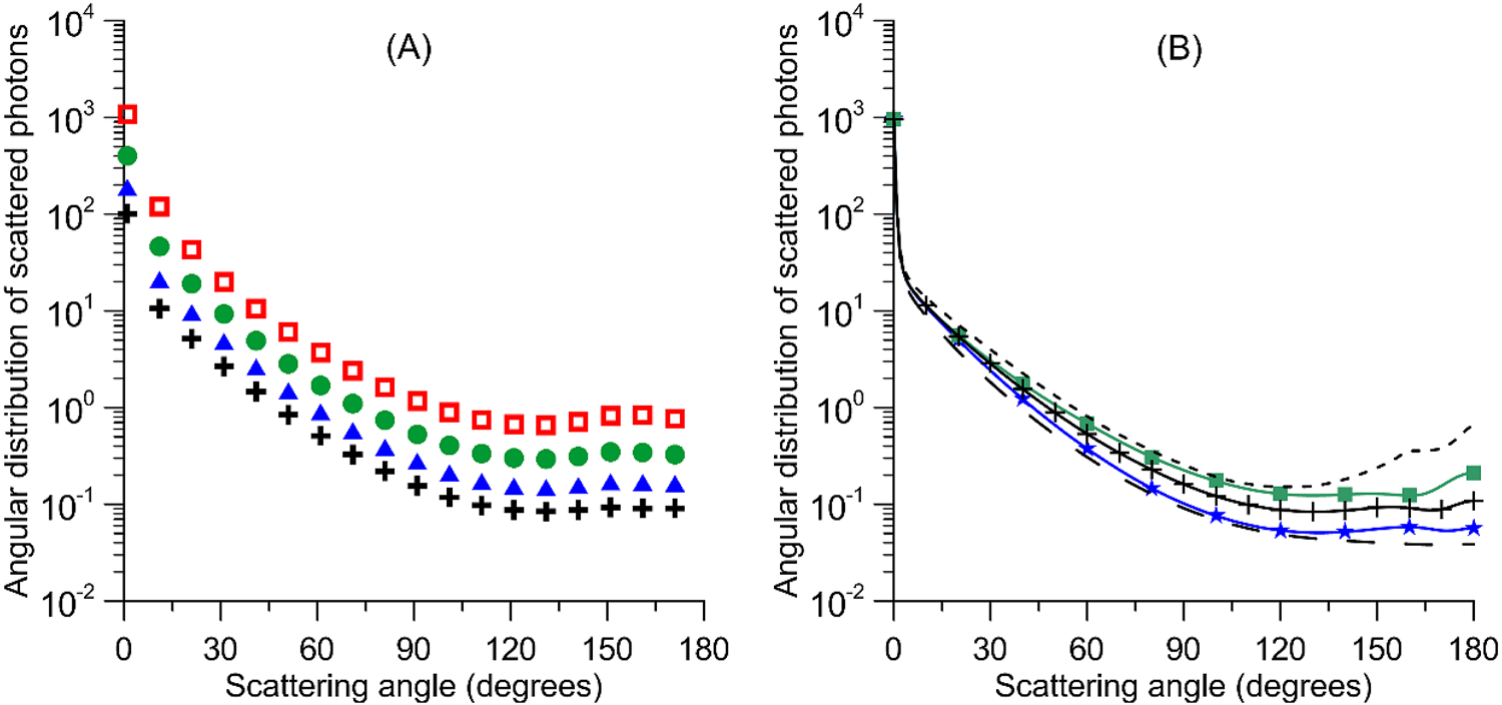
Angular distribution of scattered light intensity for light of wavelength (λ) 530 nm computed for a mixture of urban-dominated aerosol components (details are discussed in “Methods”). The number of scattered photons proportional to the differential scattering cross section^39^ is shown on y-axis relative to the number concentration of urban aerosols^36^. (**A**) Black crosses in the left plot indicate results for the baseline conditions. The plot demonstrates that an increase of the particle size by 20% (solid blue triangles), 50% (solid green circles), and 100% (hollow red squares) over baseline while holding the number concentration constant enhances the scattered light intensity in all directions. (**B**) The range of backscattering features due to the varying complex refractive index ($m=n+i\kappa$). The baseline conditions are for a mean refractive index $m$ = 1.5 + 0.05i (solid black line with cross symbols). The scattered intensity drops when lowering $n$ to 1.4 (blue stars) and increases when $n$ is raised to 1.6 (green squares). Highly absorbing elements in the aerosol mixture flatten the scattering phase function at large angles (long dashed black line for $\kappa$ = 0.2), which is contrasted by the results for non-absorbing particles (short dashed black line for $\kappa$ = 0.0).

Of various aerosol properties, the wavelength-dependent aerosol optical depth (AOD) is known to correlate well with the total concentration of particles in the air^14^. AOD is commonly used to characterize the attenuation of light beams as they traverse the atmosphere, and it relates to the cross section of particles in a particular way. We therefore expect a diversity of optical effects with a wide range of amplitudes from aerosol mixtures suspended in air. To demonstrate this effect, we simulated the angular distribution of the intensity of light scattered by a mixture of aerosol components characteristic of the air over cities (Fig. 1). Even for the same number concentration of aerosol particles, the figure shows that the number of photons scattered toward the ground can vary over an order of magnitude or more for particle populations that differ significantly in size distribution or chemical composition. By increasing the size of particles we simulate the range of changes to the NSB that we may expect from processes of particle coagulation or aggregation (see Fig. 1A). The aggregated particulate materials can also differ in chemical composition and mean refractive index, resulting in a wide range of backscattering features (Fig. 1B).

The intensity of scattered light as a function of particle size changes steeply when transitioning from the small-particle limit to its large-particle counterpart^15,16^. Scattered intensity from very small particles is proportional to the sixth power of the particle size, but particles that are large compared to the wavelengths of light typically scatter proportionally to their geometric cross section. The composition of particles relates to their complex refractive index, which in turn predetermines the phase shift or amplitude decay that a light ray suffers when passing through a particle. The latter phenomenon, in combination with the effect of different particle sizes, causes the total amount of power scattered by a particle in different directions to increase or decrease due to constructive or destructive interference. Therefore, the probability that photons will traverse specific angles after a scattering event is expected to vary largely according to the aerosol population.

Aerosols produced by nearby sources typically dominate local air pollution because the majority of the particles settle to the ground during long-range transport from more distant sites^17^. Excellent filtration and proper regulation of local air pollution sources is therefore an effective way to better control the particulate matter in a local atmosphere. For instance, the size of aerosol particles and the magnitude of AOD in the city of Bratislava, Slovakia, are both large compared to what is observed in Vienna, Austria^18^, although these cities are separated by only ~ 55 km and thus share local weather conditions in common. A modification of the light-scattering properties of the local atmosphere through active intervention may therefore have great potential as a new tool to control NSB beyond the limits achieved by streetlight modernization. This is particularly true for air-pollution treatments in developing regions with many local pollution sources such as heavy industrial activities.

To verify this hypothesis we performed detailed computations of NSB using a highly accurate model that includes the contributions to night sky radiance from many orders of scattering^19^. We performed simulations for an observer located at a range of distances from ~ 1 km to approximately 15 km from a light source. For all computed models, the peak contributions from individual scattering orders to the total diffuse irradiance at ground level are identified at 15 km from the source of light, and they do not exceed 18% for second-order scattering, 4% for third-order, 0.5% for fourth-order and 0.06% for fifth-order. The contribution of scattering orders higher than first to skyglow in a polluted atmosphere is a strong function of radial distance from the light source and tends to increase with optical path length. Therefore, the above values we found for an observer at a distance of 15 km will be significantly reduced closer to the light source. Other modeling results are available in the Supplementary Information (Supplementary Information Fig. S1).

The computations are reported relative to baseline conditions characterized by AOD_530nm_ = 0.3, aerosol Ångström exponent α = − 1.3, aerosol asymmetry parameter ASY = 0.6, aerosol single scattering albedo SSA = 0.95, and aerosol scale height ASH = 2.2 km. These values are representative of typical urban atmospheres^20^ for a nominal wavelength of 530 nm, simulating average conditions for the middle of the visible spectrum. 530 nm was chosen in particular because it approximates the peak wavelength sensitivity of the Sky Quality Meter, a widely used, commercially available NSB measurement device^21^. We performed similar computations for λλ = 450 nm and 650 nm, to demonstrate the aerosol impacts on NSB for the *B* and *R* bands of the Johnson-Cousins photometric system (see Supplementary Information for further details). AOD values > 0.3 for the middle of visible spectrum are very often found in Eastern Asia, especially in highly polluted areas of China^22^. Especially in Southeast Asia, AOD increased by more than 130% between 1980 and 2006, so proper control of source emissions can improve the situation significantly. Air pollution reduction programs in Chinese cities aim to lower the number concentration of particles with sizes < 2.5 μm by several tens of percent through reducing anthropogenic sulfate emissions that dominate AOD in industrial regions^23^. Although it is highly unlikely that air pollution can be eliminated completely, it is realistic to imagine that air pollution can be reduced substantially through effective public policy, and indeed it already has been in various parts of the world. It is therefore an important challenge to find out which NSB changes we may expect when transitioning from the aforementioned 'baseline' turbidity conditions to those in a cleaner atmosphere up to the theoretical limit in which AOD approaches zero.

**Figure 2:**
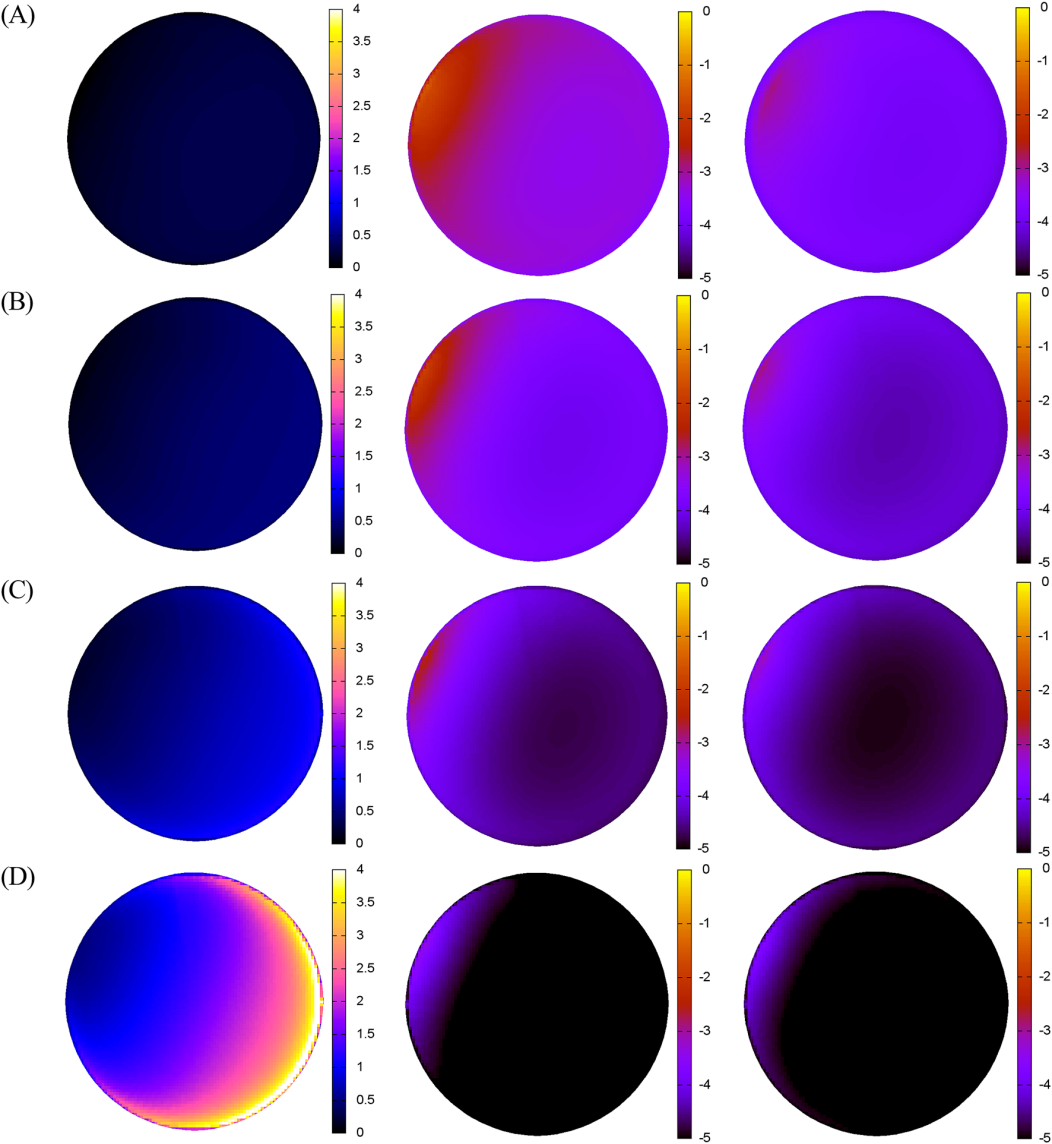
Aerosol-induced changes to NSB at a set of discrete distances, *d*, from a light source for λ = 530 nm. From (**A**) to (**D**): *d* = 1.3 km, 2.9 km, 6.7 km and 15.4 km. The leftmost column is the ratio NSB_AOD = 0_/NSB_AOD = 0.3_, shown on the same linear scale to make valid comparisons possible. The middle and rightmost columns are false-color representations of the all-sky NSB distributions (in arbitrary units on the logarithmic scale) for ‘polluted’ (AOD = 0.3) and ‘clean’ atmospheres (AOD = 0.0), respectively. The zenith and horizon are in the center and at the edge of each plot, respectively. A source of light is at an azimuth angle of 294°. The results in the leftmost column indicate that eliminating air pollution completely reduces NSB most at small distances from light sources, but at a distant place the NSB under clear-air conditions is relatively higher than that in turbid air. Far from the light source (**D**) we observe a decrease of total NSB by one to two orders of magnitude compared to what is obtained for (**A**) in the middle and rightmost columns, so the relative impact of AOD on NSB is probably not critical for either a measuring device or a human observer.

For a light source at a distance of 1.3 km from an observer emitting 10% of its photons directly upward, a change from polluted air (AOD_530nm_ = 0.3) to a clean atmosphere results in zenith NSB reduction by a factor of 3.2; i.e., NSB drops to ~ 30% of its initial level (leftmost plot in Fig. 2A). Increasing the AOD for a given fixed distance from the light source to the observer causes the flux density of the light beam to decay rapidly. Therefore, the zenith NSB in clean air conditions increases to 40% of that obtained in turbid air if the distance to the light source approaches 3 km; 60% at the distance of about 7 km; and even 140% for an observer situated 15 km from the light source (see leftmost plots in Fig. 2B–D). The NSB for truly zero AOD determined relative to NSB for AOD = 0.3 is a good indicator for air pollution control policy impacts; however, an inverse relationship between zenith NSB_AOD = 0_/NSB_AOD = 0.3_ and the ground distance from the light source should be interpreted in context along with the significantly reduced number of photons detected in the zenith at large source distances. As the source distance increases from 1.3 km to 3 km, 7 km, and 15 km, the zenith NSB in a turbid atmosphere falls to 26%, 5% and 0.4% of its starting value, respectively; however, the corresponding trend in a clean (‘Rayleigh’) atmosphere is less pronounced, with corresponding rates of 33%, 9%, and 2%. So even if the ratio NSB_AOD = 0_/NSB_AOD = 0.3_ in the zenith tends to increase with source distance, the amplification factor of 1.4 found for the distance of 15 km from the light source is negligible compared to the general exponential decay of NSB values with increasing distance. In a clean atmosphere the total sky brightness in the zenith 15 km from the source of light is 50 times smaller than that obtained at a distance of 1.3 km. Therefore, an increase of NSB_AOD = 0_ relative to NSB_AOD = 0.3_ by a factor of 1.4 is very small in view of the low values of total NSB we found at distant places compared to those near the light source. A large decrease in NSB from backscattered light is noticed in the sky direction opposite to the azimuthal direction of the light source (see middle and rightmost columns in Fig. 2).

It is evident that air pollution control critically influences NSB near a light source, while potentially increased values of NSB_clean-air_/NSB_turbid-air_ far from a light source are not a sufficient reason to invoke actions to mitigate NSB. The findings are important for observatories near or even inside cities very often intended for astronomy education and/or public outreach, like those traditionally located in the peripheries of European cities. In professional astronomy, narrow-band photometry suppresses the negative impacts of light source spectral power distributions prevailing on local scales; however, enhanced emissions in blue wavelengths, predominately from white LED sources, may induce significant impacts to visual astronomy^9^ (see Extended Data Figs. S2, S3 for blue light and red light).

**Figure 3:**
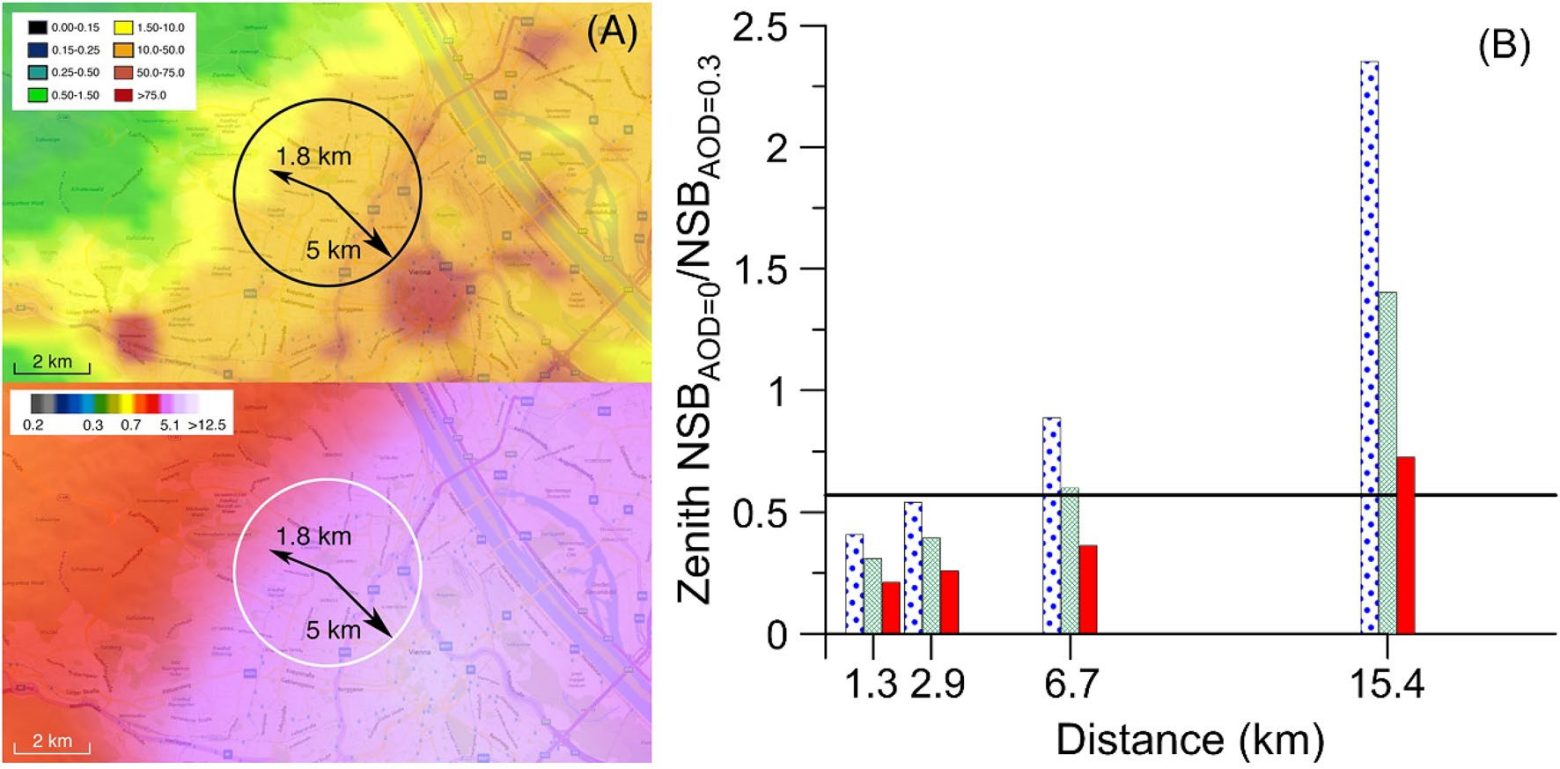
Sources of artificial light near the Vienna measuring station and their modeled NSB impact as a function of AOD and distance. (**A**) Situation of the University of Vienna Institute for Astrophysics (IFA) with respect to the spatial distribution of upward radiance and zenith night sky brightness over the city of Vienna, Austria. In the top panel, the upward-directed radiance in nW cm^−2^ sr^−1^ is shown in false colors according to the key at upper left, overlaid on a background map of Vienna. The zone defined by the 5-km circle encloses light sources with the highest relative importance for the experimentally determined ratio of NSB_AOD = 0.06_/NSB_AOD = 0.4_ discussed in the main text. In the bottom panel, the same information is shown but the false colors indicate the predicted zenith night sky brightness from the maps of Falchi et al.^1^. The scale at upper left shows the zenith brightness in units of mcd m^−2^. (**B**) Model predictions of the NSB_AOD = 0.06_/NSB_AOD = 0.4_ ratio as a function of light source distance from the Vienna measurement station. The ratios computed are shown as bars with blue dots for wavelengths of 450 nm, bars with green crossed lines for 530 nm, and red bars for 650 nm. The horizontal black line indicates the measured NSB_AOD = 0.06_ /NSB_AOD = 0.4_ NSB ratio (0.57).

To test our hypothesis concerning the link between air pollution and skyglow, we validated the predictions of our models using experimental data obtained in short-term circumstances simulating the systematic reduction of air pollution. For this experiment we identified two dates in spring 2019 when local weather conditions over the city of Vienna, Austria, allowed for the comparison of nighttime conditions during which AOD was relatively high and low. AOD_500nm_ measured at the AERONET (aeronet.gsfc.nasa.gov) station in Vienna declined rapidly during this period, from 0.4 on 6 April to 0.06 on 9 April. The position of the Institute for Astrophysics (IFA) measuring station relative to the center and edges of the Vienna conurbation is shown in Fig. 3A. The 5-km vector indicates the radial distance from IFA to the Vienna city center, and the 1.8-km vector represents the mean distance to the continuously built edge of the city. The data shown in the top panel represent the March 2019 monthly cloud-free composite radiance obtained from remote sensing measurements made by the Visible Infrared Imaging Radiometer Suite in its Day/Night Band^24,25^. The map follows the usual cartographic convention of north at top and east at right, and a 2-km scale bar is provided in the lower left corner. The zenith night sky brightness shown in the bottom panel are taken from lightpollutionmap.info (Map by Jurij Stare; Microsoft product screen shot reprinted with permission from Microsoft Corporation).

These two dates in April 2019 were chosen not only for their large difference in AOD, but also because the other aerosol parameters varied only slightly between them: the single scattering albedo (SSA) was 0.98 on the first date and 0.97 on the second date; the absorption AOD was 0.006 on both dates; and the aerosol asymmetry parameter (ASY) changed from 0.71 to 0.61. We measured the change in zenith NSB on the two nights at the same time shortly before local midnight using a Sky Quality Meter operated at IFA (https://astro.univie.ac.at/ueber-uns/lichtverschmutzung/). During the high-turbidity event on 6 April it was (4.30 ± 0.12) mcd m^−2^, while under clear-air conditions on 9 April it was (2.47 ± 0.07) mcd m^−2^. The ratio of the zenith brightness values, NSB_AOD = 0.06_/NSB_AOD = 0.4_, was therefore 2.47/4.30 = 0.57 ± 0.04. To estimate the uncertainty, we used the known instrumental scatter of the SQM device, which averages to about ± 3% (± 0.03 magnitudes per square arcsecond).

We computed models of NSB for the same distances of *d* = 1.3 km, 2.9 km, 6.7 km and 15.4 km as shown in Fig. 2 and compared the predicted values of NSB_AOD = 0.06_/NSB_AOD = 0.4_ against our measurement to find the mean distance to light sources contributing the most NSB light at the zenith. Figure 3B shows the NSB ratios for the selected light source distances along with our measured value from the IFA station. The horizontal black line shows our measured NSB ratio (0.57), which is best matched by the model for which *d* = 6.7 km. The figure therefore suggests that our measured value is theoretically due to light sources at distances ≲ 7 km, and is mostly attributable to light sources distributed within the mean radius of 5 km shown in Fig. 3A.

While we are unable to determine exactly which light sources dominate the contribution to zenith NSB at the IFA station, certainly some of those sources are within a wider interval of radii, *R*. The number of light sources in a city, *n*, increases roughly as the square of the distance from a given location (*n* ~ *R*^2^), so we conclude that light sources in the neighborhoods nearest IFA alone cannot be decisive for its NSB. Light from sources at smaller distances contributes efficiently to the total brightness of the zenith sky because the relatively shorter optical paths of those light rays mean that they suffer less atmospheric attenuation from source to observer. However, as noted previously, there are few such sources at short range from IFA. Distant sources (*d* > 7 km) contribute less efficiently to zenith brightness at the IFA because of the longer optical paths of their light rays, but their numbers are relatively high.

The most significant contribution to NSB at this site is from light sources in some radius interval *R*_1_ to *R*_2_ because of two opposite effects. First, as noted above, *n* is a quadratically increasing function of *R*. However, because the radiance of a light source varies as *R*^−2^, and in consideration of the exponential attenuation of the light beam along its direction of travel, the real optical signal varies as *R*^−*N*^, where *N* > 2 and depends on factors such as the atmospheric turbidity and the length and inclination angle of the beam path. We therefore expect that the weighted contribution of nearby light sources is higher than that for distant sources, and that the contribution to the NSB is mostly from lights at *d* ≤ 7 km. For larger values of *d*, the contribution from light sources to the NSB is expected to decrease steeply^26^.

**Figure 4:**
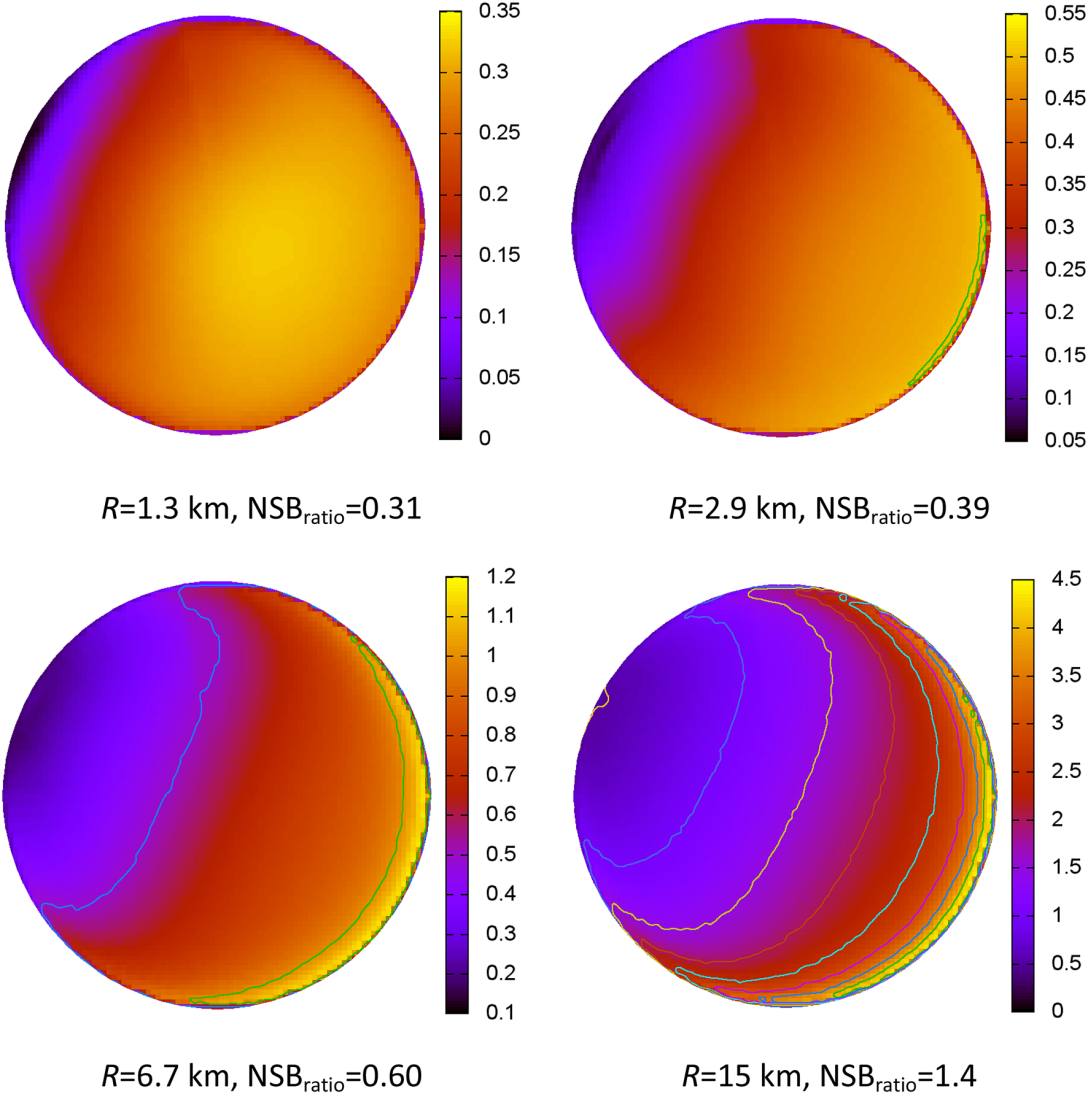
Model predictions of the NSB_AOD = 0_/NSB_AOD = 0.3_ ratio for point light sources at the indicated distances. The other model input parameters (α = − 1.3, ASY = 0.6, SSA = 0.95 and ASH = 2.2 km) were the same among all four models. These plots match the orientation of those shown in the leftmost column of Fig. 2, but the scales are different in each panel. To emphasize the details in each case, we have scaled them differently in order to adapt the dynamic range of the NSB ratios shown.

We computed generic models of point light sources at the same fiducial distances as before, which are shown in Fig. 4. The models have identical values of ASH (2.2 km) and ASY (0.6). We allowed the NSB_AOD = 0.3_/NSB_AOD = 0.0_ ratio to vary according to the values shown by the green bars (λ = 530 nm) in Fig. 3B. Although the measured ΔAOD (− 0.34) is different from the ratio used to compute these generic models (− 0.30), we find good correspondence between the model predictions and our IFA observations.

Formally speaking, our model should be tested in the context of a deliberate, persistent reduction in air pollution. Even though we only showed the result for a case in which weather effects simulated a systematic pollution reduction, the test performed is appropriate to validate the model. For as long as air pollution levels are reduced, whether temporarily (in the case of Vienna) or permanently (through effective policy changes), the effect should be the same because the underlying physics is the same. Although we looked at one instance in which certain aerosol properties changed in a particular way, the combination of parameters resulting in a decrease in AOD is less important; furthermore, in a cleaner atmosphere the aerosol properties would not be determinative of the result since NSB asymptotically approaches that of constant background for Rayleigh scattering. We therefore expect that the conclusions reached here will be equally applicable to any situation in which atmospheric AOD is reduced for whatever reason.

## Methods

### The clear sky atmospheric model

It is a crucial determinant of reliable NSB predictions to adopt a cloud-free atmospheric model that is very closely aligned with conditions observed in nature. In practice, the main endeavor of the model is to cover fundamental aspects of atmospheric optics while keeping the approximations imposed on the model parameters and properties within the range of values and trends commonly observed in the atmosphere. Any such model must have a theoretical foundation providing satisfactorily accurate predictions; make no extreme demands on computational resources; and allow for real-time numerical experimentation. The multiple scattering solver^19^ designed for NSB characterization is briefly described in “Determining higher-order scattering radiances” section below, except for its properties and limits which are both summarized here.

Due to the relatively short distances from a light source to the observer in the case of skyglow seen over a city, radiative transfer is confined here to the case of a plane-parallel atmosphere characterized with a good horizontal uniformity of its composition. We make this standard simplification as it was found reliable in radiative transfer modeling under clear sky conditions and it reduces the model’s complexity and streamlines the computations while giving proper attention to important physical aspects of the problem to be solved when the atmosphere is of finite optical thickness. The latter is important in order to choose a suitable method to solve the radiative transfer equation (RTE)^27^. The atmospheric optical depth can scarcely exceed unity under clear sky conditions, thus allowing use of the Method of Successive Orders, which is simple in concept; fast in convergence; easy in understanding of the underlying physics; and straightforward in the interpretation of optical phenomena due to predictable photon paths.

We model Earth’s atmosphere as an anisotropic scattering medium containing randomly oriented scatterers. Random orientation is of especially high relevance to aerosol particles, which can be large in size compared to the wavelength of visible light and thus exhibit various scattering patterns depending on their instantaneous orientation relative to the direction of the incident light. Particles with preferred orientations show very distinct scattering signatures that would be extremely difficult to incorporate into a common model, but random orientations allow for ensemble averaging and for use of approximate formulae such as the Henyey-Greenstein scattering phase function ${P}_{a}\left(\theta \right)=\left(1-{g}^{2}\right)/{\left(1+{g}^{2}-2g{\cos}\theta \right)}^{3/2}$. Here the asymmetry parameter ($g$) is known to range from − 1 to 1, while favoring forward-lobed ${P}_{a}\left(\theta \right)$ (i.e., photons directed preferentially to scattering angles $\theta <90^\circ$) in the case of large particles and transitioning to an intensity distribution that is symmetric in both the forward and backward directions as $g$ approaches zero.

In our clear-sky atmospheric model we account for scattering and absorption by aerosol particles and Rayleigh scattering by air molecules. The attenuation due to absorption bands of atmospheric gases is neglected because of short optical path lengths between light source and observer. We know from extensive numerical modeling^28^ that the contribution to NSB from multiple scatterings increases with ground albedo, especially in the direction opposite to the azimuthal position of the light source. Although the ground albedo is generally low when snow cover is absent, in this paper we model sky brightness from the superposition of five higher-order radiances in order to guarantee the NSB is accurate to within a specified error tolerance. The solution to the monochromatic RTE allows the simulation of both narrowband (quasi-monochromatic) and broadband radiances. We do not consider the polarization state of ground-reaching radiation because most devices employed in night-sky monitoring are designed to average intensity measurements irrespective of polarization. We base our assessments on a well-defined restriction to the unpolarized RTE along with the fact that light emerging from artificial sources is typically unpolarized.

To interpret the bulk optical properties of the atmospheric environment we use the following five aerosol parameters: (i) asymmetry parameter ($g$) also called ASY throughout this paper; (ii) single scattering albedo (SSA); (iii) optical depth at a reference wavelength of 500 nm (AOD_500nm_); (iv) Ångström exponent (α); and (v) aerosol scale height (ASH). Extremely low values of ASY are rare among distinct aerosol populations. Most studies suggest ASY > 0.5, frequently grouped around the value of 0.6^29^. SSA, which characterizes the fraction of electromagnetic energy scattered relative to that removed from an incident beam of light, is generally large except for highly absorbing materials. The value of SSA = 0.95 we used in our modeling is well within the range of data displayed ibidem. The Ångström exponent models the spectral behavior of AOD in the form AOD = AOD_500nm_ × (λ/500 nm)^α^, where λ is in nanometers. Horvath et al.^20^ found that α is close to − 1.5 but normally spans a wide interval of values from − 2 to 0 or even more. We used α = − 1.3 in the numerical experiments reported here along with ASH = 2.2 km^30^. We varied AOD_500nm_ in our numerical models, and the respective values used are indicated in the figure captions. In most of cases AOD_500nm_ = 0.32 (i.e., AOD_530nm_ = 0.3 for α = − 1.3) unless explicitly defined otherwise. The Rayleigh optical depth has been computed according to the method of Duan, Min, and Stamnes^31^.

### Computing optical properties of urban aerosols

While the composition and size distribution of aerosols are subject to large spatial variations, aerosol types are generally classified according to their characteristics. Aerosol type classifications are geographically specific, except for situations in which the particles underwent long-range transport (as can be observed, e.g., during dust events^32^). We can therefore distinguish between and among urban aerosols, rural aerosols, maritime aerosols, etc. In rural settings, aerosols tend to be a mixture dominated by water-soluble solids with the addition of some dust-like particles^33^. Urban aerosols are similar to rural aerosols, but industrial and automotive activities replace a minority of rural-type particles with soot-like aerosols. In the computation of our models for Vienna, we adopted an aerosol composition consisting by volume of ~ 50% ammonium sulfates, ~ 40% organic matter, and ~ 10% black carbon with mean complex refractive index $m$ = 1.5 + 0.05*i* obtained according to the Bruggeman mixing rule^34^. The value of $m$ slightly differs from that obtained in Kocifaj, Horvath and Gangl^35^.

The choice of a particular aerosol model is based on the target simulation. We selected an urban aerosol model because most light emitted upwards originates from human settlements. However, rural and urban aerosol models do not differ much in size distribution, so the modeling results appear relevant for large cities and smaller towns as well. The aerosol number distribution is modeled by the bimodal lognormal function $f\left(r\right)=\frac{dN\left(r\right)}{dr}\propto \sum_{i=1}^{2}\frac{{N}_{i}}{r{ \sigma }_{i}}{\exp}\left[-\frac{1}{2}{\left(\frac{{\log}r-{\log}{r}_{i}}{{\sigma }_{i}}\right)}^{2}\right]$, where $N\left(r\right)$ is the number of particles having a radius between $r$ and $r+dr$. The numerical constants used to model the baseline conditions were as follows: ${N}_{1}$ = 0.999875, ${N}_{2}$ = 0.000125, ${\sigma }_{1}$ = 0.35, ${\sigma }_{2}$ = 0.4, ${r}_{1}$ = 0.06 µm, ${r}_{2}$ = 1.17 µm^36^. We were inclined to accept model values for elevated relative humidity conditions to be in line with the fact that some constituents, such as large, dust-like aggregates or water soluble aerosols, can remain suspended in the atmosphere for a longer time. The angular distribution of the scattered light intensity relative to the number concentration of urban aerosols displayed in Fig. 1 is computed from $\left\{{\int }_{0}^{\infty }f\left(r\right)\frac{{i}_{1}\left(\theta ,r\right)+{i}_{2}\left(\theta ,r\right)}{2}dr\right\}/{\int }_{0}^{\infty }f\left(r\right)dr$, where ${i}_{1}$ and ${i}_{2}$ are intensity distribution functions determined as the squares of Mie amplitude functions ${S}_{1}$ and ${S}_{2}$, respectively^37^.

### Determining higher-order scattering radiances

Radiative transfer in a plane-parallel atmosphere reduces to an integro-differential equation that can be solved iteratively subject to homogeneous boundary conditions^38^. The iterative procedure is straightforward for an atmosphere illuminated uniformly from below; however, the physical model for first-order radiance requires special treatment in cases where the uniformly bright interface is replaced by an array of isolated sources emitting light arbitrarily into the upper hemisphere. Since the eigenvalues are low for a cloudless atmosphere, we have identified no convergence problems even for extreme values of AOD_500nm_ ≈ 0.6–0.8 and even for elevated ground albedo values (not shown in this paper).

The first-order downward and upward radiances due to light emissions from a single source have been derived by Kocifaj^19^ in the form of so-called generating functions. The higher-order radiance formalism is to generate *j*-th radiance field out of (*j* − 1)-th radiance field. The latter is assumed to be known for all grid points. For a plane-parallel, exponentially stratified atmosphere we preferred to integrate over the optical depth ${\tau }{^{\prime}}$ rather than altitude, so the downward radiance at the optical depth $\tau$ in the *j*-th scattering order approximation is

1 $${I}_{j}^{+}=\frac{1}{\mu }\underset{{\tau }{^{\prime}}=0}{\overset{\tau }{\int }}{\exp}\left(\frac{{\Delta \tau }}{\mu }\right)\left\{{\int }_{+}{I}_{j-1}^{+}\frac{{p}^{+}}{4\pi }d{\Omega }^{{\prime}}+{\int }_{-}\left[{I}_{j-1}^{-}+\frac{a}{\pi }{\exp}\left(\frac{\Delta {\tau }_{0}}{\mu }\right){D}_{j}^{+}\right]\frac{{p}^{-}}{4\pi }d{\Omega }{^{\prime}}\right\}d\tau {^{\prime}}$$

where ${I}_{j-1}^{+}$ and ${I}_{j-1}^{-}$ are the downward and upward radiance fields at the altitude corresponding to the actual value of ${\tau }{^{\prime}}$; ${D}_{j}^{+}$ is the total diffuse irradiance at the ground; ${p}^{+}$ and ${p}^{-}$ are the scattering phase functions for light beams traversing the atmosphere from a direction ${{\varvec{\Omega}}}^{\boldsymbol{^{\prime}}}=\left({\mu }{^{\prime}},A{^{\prime}}\right)$ towards ${\varvec{\Omega}}=\left(\mu ,A\right)$. Here ${\mu }{^{\prime}}={\cos}z{^{\prime}}$, where ${z}^{\prime}$ is the zenith angle and $A{^{\prime}}$ is the azimuth angle, both of which are used as local variables in numerical integration. Analogously $(\mu ,A$) defines the direction of light beam after the *j*-th scattering event. The first integral on the right-hand-side of Eq. (1) is taken over the upper hemisphere (+), while the symbol (−) in the second integral indicates the integration of all light signals entering the atmospheric layer $\tau$ from below. The optical depth $\tau$ ranges from 0 (at the top of atmosphere) to ${\tau }_{0}$ (at the ground), while ${\Delta \tau }={\uptau }{{^{\prime}}}-\uptau$ and $\Delta {\uptau }_{0}={\tau }^{{\prime}}-{\tau }_{0}$. All functions/parameters including ground albedo ($a$) are considered to be wavelength-dependent. Exact expressions for the above functions and for the upward radiance can be found in the previous work of Kocifaj^19^.

The total radiance is obtained by summing over all computed scattering components: *I* = $\sum_{j}{I}_{j}^{+}$. In the numerical experiments in this paper, the index *j* floats from 1 to 5. The diffuse irradiance is determined from ${D}_{j}^{+}={\int }_{0}^{2\pi }{I}_{j}^{+}\mu {^{\prime}}d {\Omega {^{\prime}}}$. Due to their exponential stratifications the concentrations of atmospheric constituents and the integration along the vertical direction are both replaced in Eq. (1) by a more convenient approach based on the total optical depth. The latter also changes roughly exponentially, so we can avoid dynamically adapted lattice spacing along the vertical direction and keep the integration error low for relatively low numbers of grid points. The integration error is controlled by adjusting the integration step to half of its initial value.

## Supplementary Information

Supplementary Information.

## Data Availability

The experimental and computational data are available at the IDA and Slovak Academy of Sciences (web-page). The other data that support the results of this study are available from the corresponding author upon reasonable request. (Extended data is available for this paper). Access to the multiple scattering code used in this work is available from the authors upon request.
