# Supplementary material for: Air pollution mitigation can reduce the brightness of the night sky in and near cities
Source: Sci Rep. 2021 Jul 16;11:14622. doi: 10.1038/s41598-021-94241-1 (PMC8285390; doi:10.1038/s41598-021-94241-1)
Supplement: Supplementary file 1 — Supplementary Information. [file 41598_2021_94241_MOESM1_ESM.docx]

Air pollution mitigation can reduce the brightness of the night sky in and near cities

Miroslav Kocifaj^1,2^* and John C. Barentine^3,4^

^1^ICA, Slovak Academy of Sciences, Dúbravská Road 9, 845 03 Bratislava, Slovakia

^2^Faculty of Mathematics, Physics, and Informatics, Comenius University, Mlynská Dolina, 842 48 Bratislava, Slovakia

^3^International Dark-Sky Association, 3223 N. First Avenue, Tucson, AZ 85719 USA

^4^Consortium for Dark Sky Studies, University of Utah, 375 S 1530 E, RM 235 ARCH, Salt Lake City, Utah 84112-0730 USA

*Correspondence to: e-mail.

**Supplementary Information**

The numerical experiments were conducted to demonstrate that spectral horizontal irradiances determined from the superposition of five scattering orders are highly accurate even in polluted atmospheres with a maximum contribution from fifth order not exceeding 0.1% of the total irradiance for blue light (λ = 450 nm, AOD_450nm_=0.37), 0.06% for green light (λ = 530 nm, AOD_530nm_=0.3), and 0.02% for red light (λ = 650 nm, AOD_650nm_=0.23), as shown in the left column in Figure S1. The discrete wavelengths are the same as in Figure 3B. We also found that the spectral sky radiance over whole upper hemisphere computed for *d*=1.3 km (upper left-hand corner in the rightmost column of Figure S1) is almost exclusively due to single scattering. The contribution from the first scattering order to the total radiance for an observer located 15.4 km from the light source (lower right-hand corner) does not fall below 0.7, and for sky elements 20-30° above horizon more than 80% of all light is due to single scattering.

The analysis of aerosol-induced changes to NSB we performed in the main text is extended to the wavelength of 450 nm in Figure S2. Blue light scatters more efficiently than other wavelengths in the visible spectrum; thus the ratios in leftmost column of Figure S2 are generally higher than those for green light (Figure 2 introduced in the main part of the paper) or red light (Figure S3, below). This is why significant NSB mitigation when comparing a turbid to an unpolluted atmosphere is most apparent in the long-wavelength domain of the visible spectrum. For instance, the zenith brightness in the blue region of the spectrum for a clean atmosphere at a distance of 6.7 km from a light source is 88% of that in turbid conditions, while the relative decrease is 60% for green light and 36% for red light in the same scenario. Therefore, an effective air pollution control policy can have important impacts on NSB mitigation primarily at short distances (up to ten kilometers from a light source), with relative benefits -32% (= $\frac{\boldsymbol{60\%-88\%}}{\boldsymbol{88\%}}$) for green light with respect to blue light, and even -60% (= $\frac{\boldsymbol{36\%-88\%}}{\boldsymbol{88\%}}$) for red/blue. The most negative relative impacts on NSB are identified for sky elements situated opposite to the azimuthal position of light source; this is a common feature for all wavelengths (but with emphasis in blue). However, for all wavelengths the NSB amplitudes decay exponentially with distance, so a relative increase of NSB_unpolluted_/NSB_turbid_ is not significant within a few kilometers of the light source.

The results for the wavelength of 650 nm (Figure S3) are important in many other aspects. Due to its weaker scattering and extinction efficiencies, red light propagates into the nocturnal environment more readily than other visible wavelengths. This is especially important for air molecules, which tend to scatter inversely proportional to the fourth power of wavelength, so the Rayleigh optical depth at 650 nm is at least four times lower than that at 450 nm. Unlike air molecules, the aerosol particles are large in size and exhibit weaker spectral dependences compared to those expected from Rayleigh theory. In our modeling experiment the Ångström exponent was as low as -1.3, while the corresponding value is -4 in the Rayleigh regime. Therefore, a relatively low aerosol concentration can cause the intensity of scattered light to increase noticeably and vice versa; therefore, as we have discussed, air pollution control can result in substantial mitigation of NSB. The transition from polluted air to a clean atmosphere is associated with NSB reduction to ~21%, 26%, 36%, and 72%, for distances of *d*=1.3 km, 2.9 km, 6.7 km and 15.4 km, respectively (see leftmost pane from top to the bottom). The positive impacts of air pollution control are identified at all distances studied, which can be of high significance not only to professional astronomical observatories surrounded by a number of localized light sources, but also those at short to intermediate distances from large metropolitan areas (such as, e.g., Leopold-Figl-Observatorium für Astrophysik at a horizontal distance of 16 km from bright edges of Vienna).

| 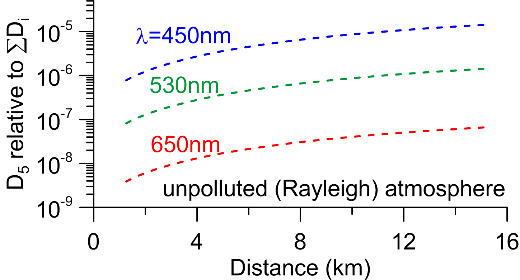  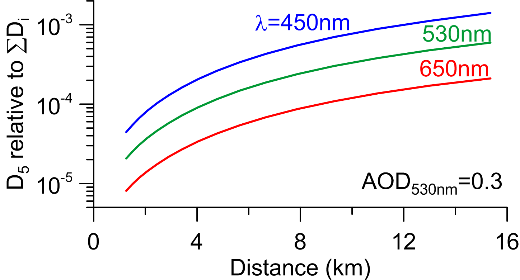 | 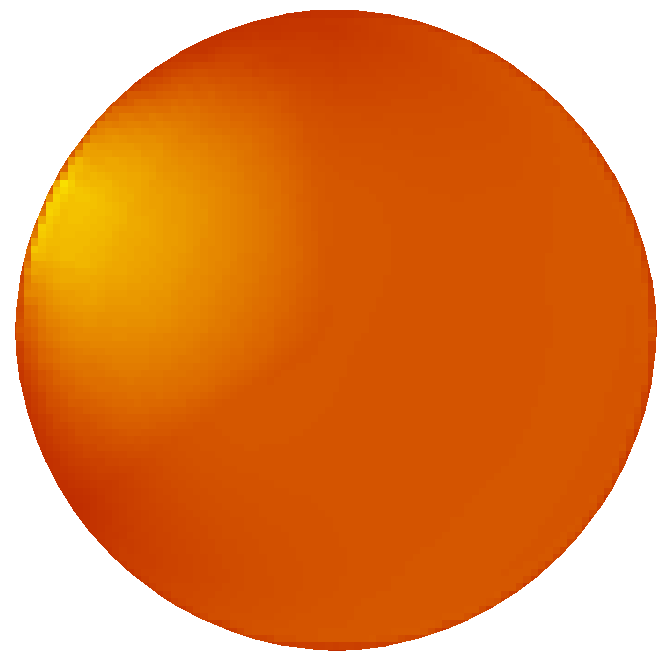 | 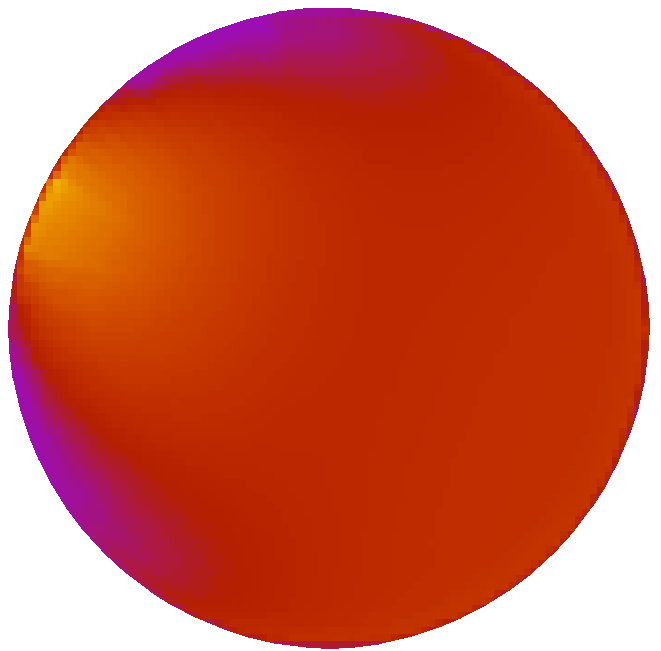 | 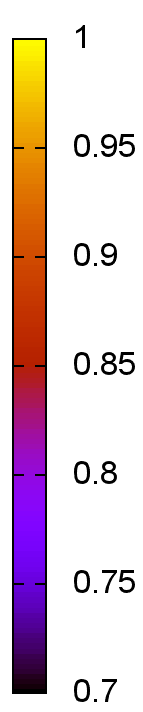 |
| --- | --- | --- | --- |
|  | 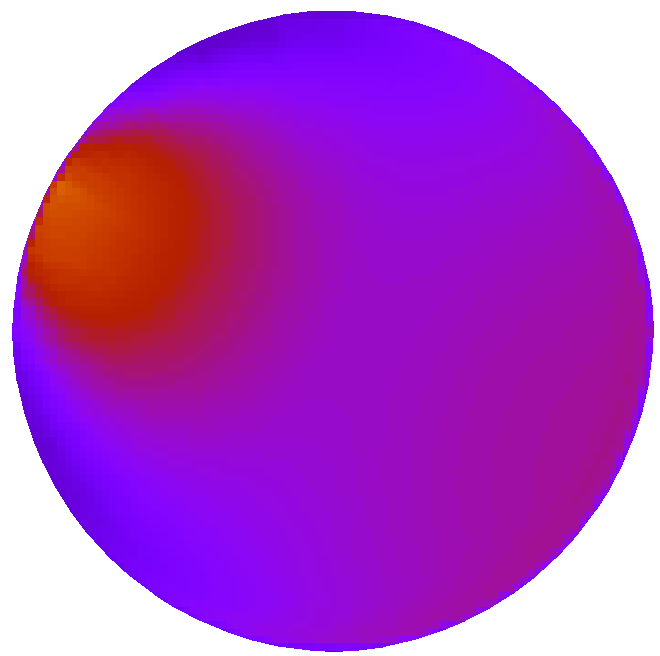 | 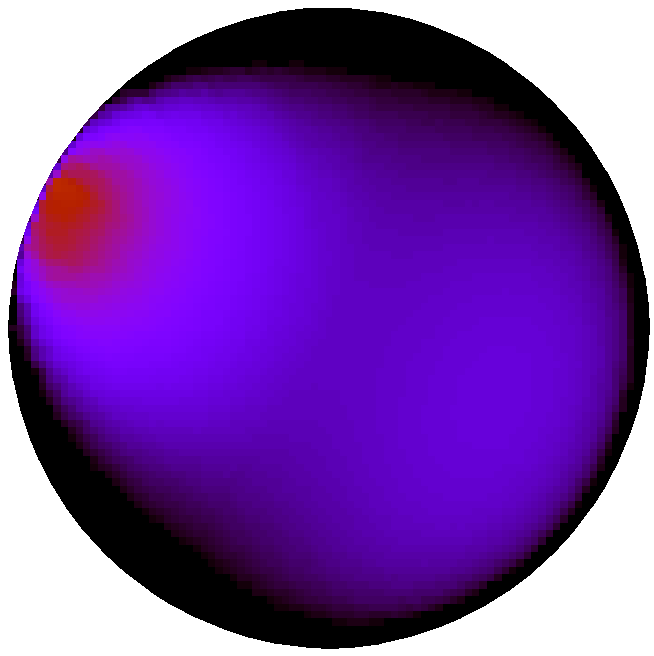 |  |

**Fig. S1:** **Numerical accuracy tests for irradiance and radiance computations using a multiple scattering code limited to five scattering orders.** The left column documents the error margin of irradiance computations by relating the contribution from the fifth scattering order to the total irradiance at a set of discrete distances, *d*, from a light source. The upper plot is for an unpolluted atmosphere (AOD_500nm_=0.0), while the bottom plot is for AOD_500nm_=0.32 and Ångström exponent α=-1.3 (implying that AOD_530nm_=0.3). Each of the four all-sky false-color images organized in the 2×2 array at the right-hand side of the figure displays models of the contribution from the first scattering order to the total radiance ($I_{1}/\sum_{j=1}^{5} I_{j}$) for green light (λ = 530 nm). From left to right and from top to bottom: *d*=1.3 km, 2.9 km, 6.7 km and 15.4 km.

| (A) | 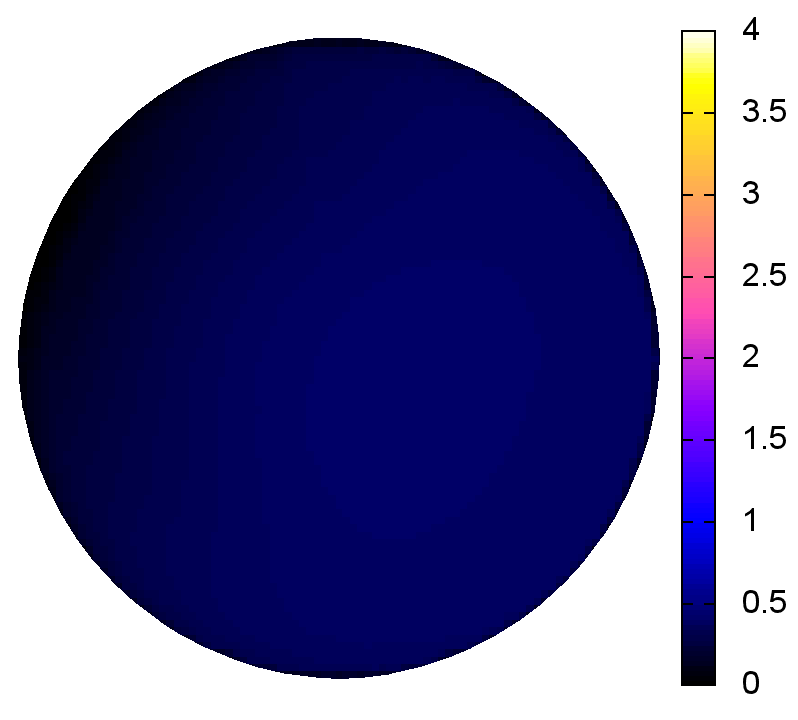 | 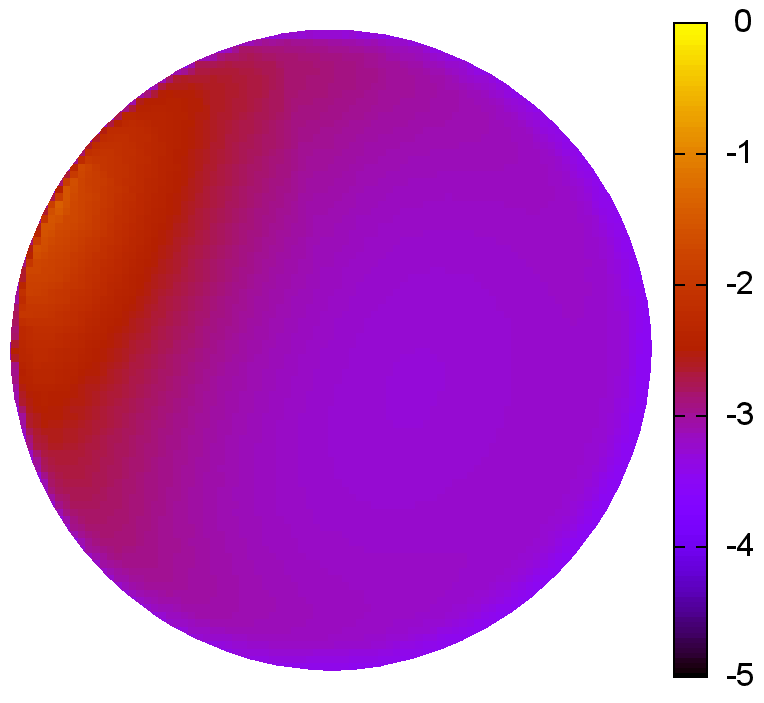 | 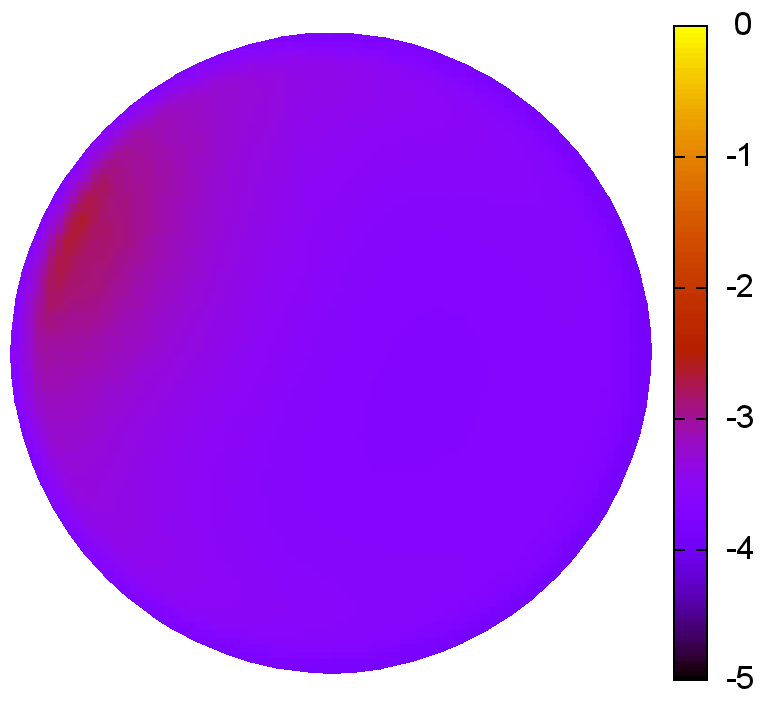 |
| --- | --- | --- | --- |
| (B) | 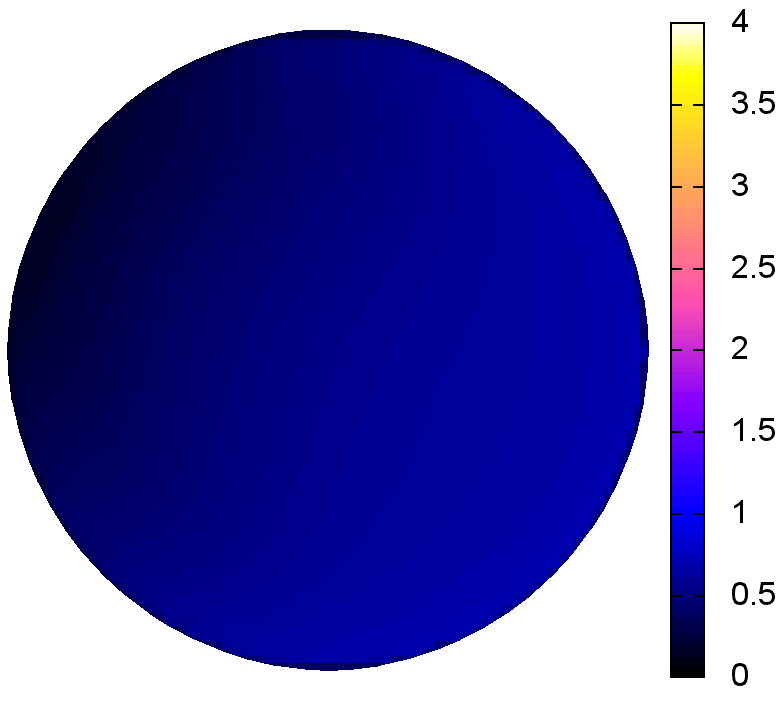 | 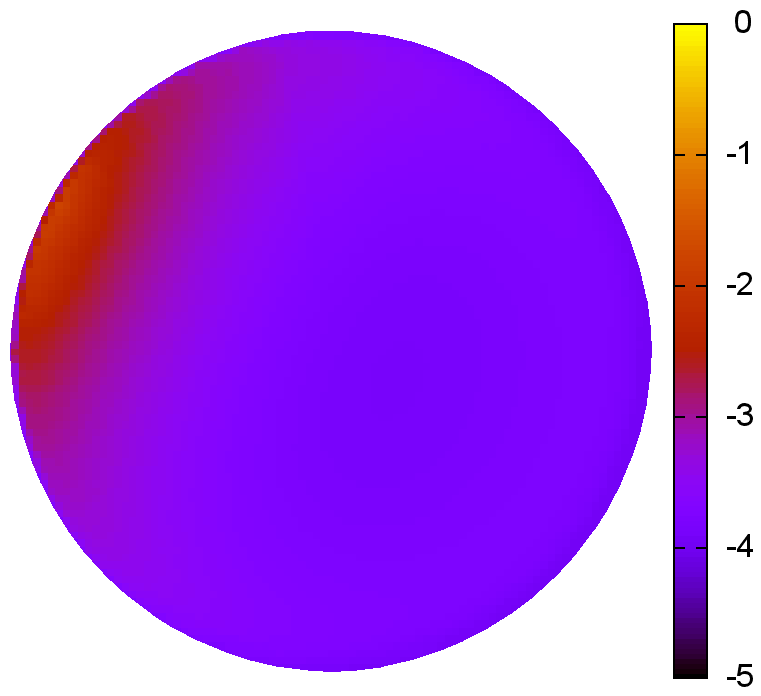 | 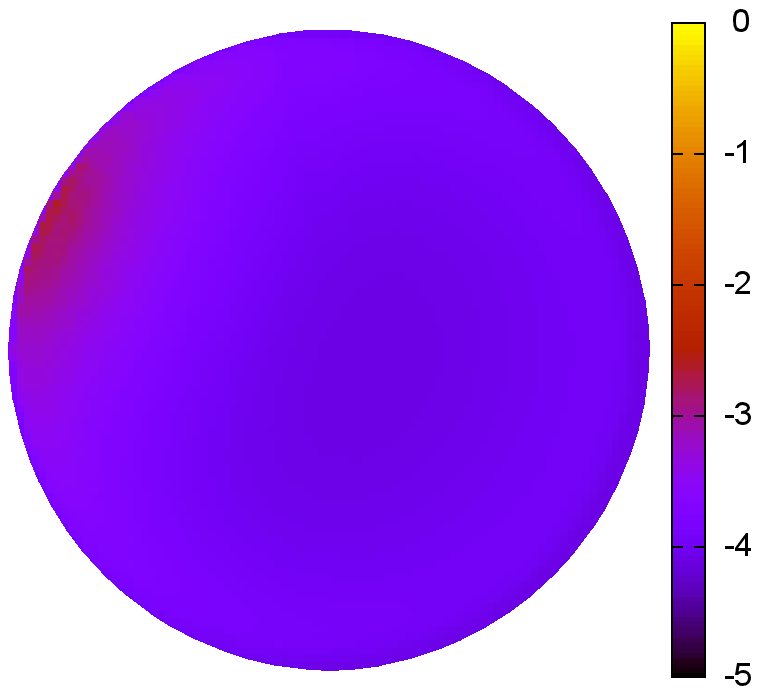 |
| (C) | 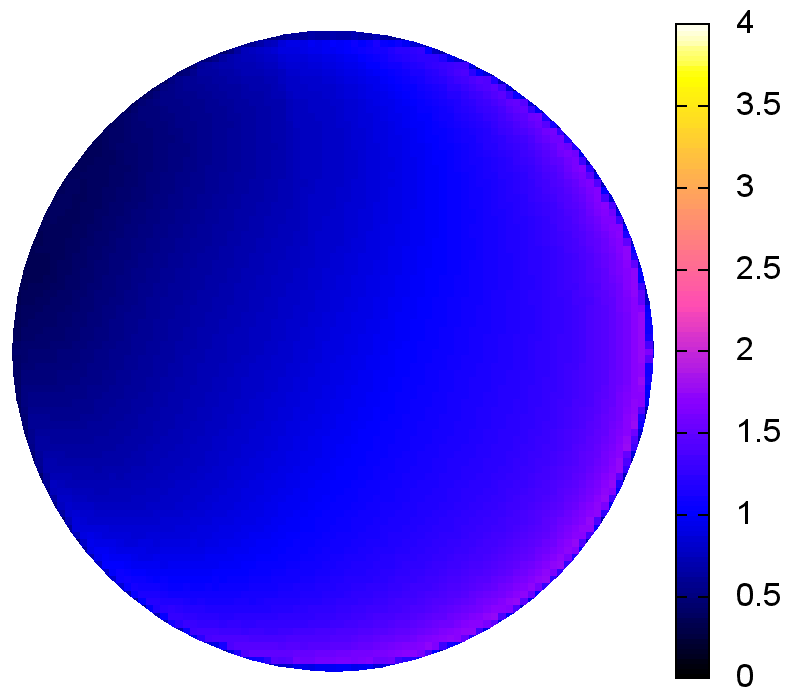 | 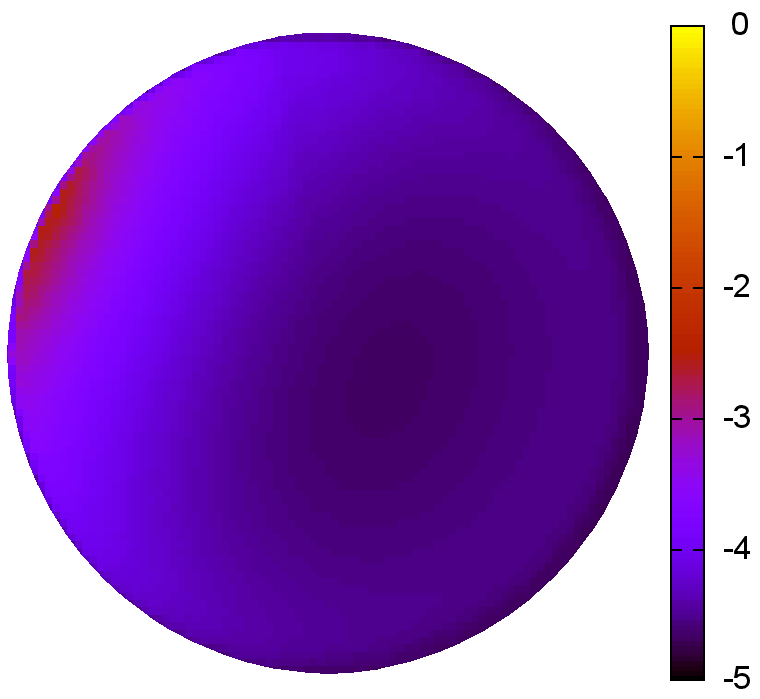 | 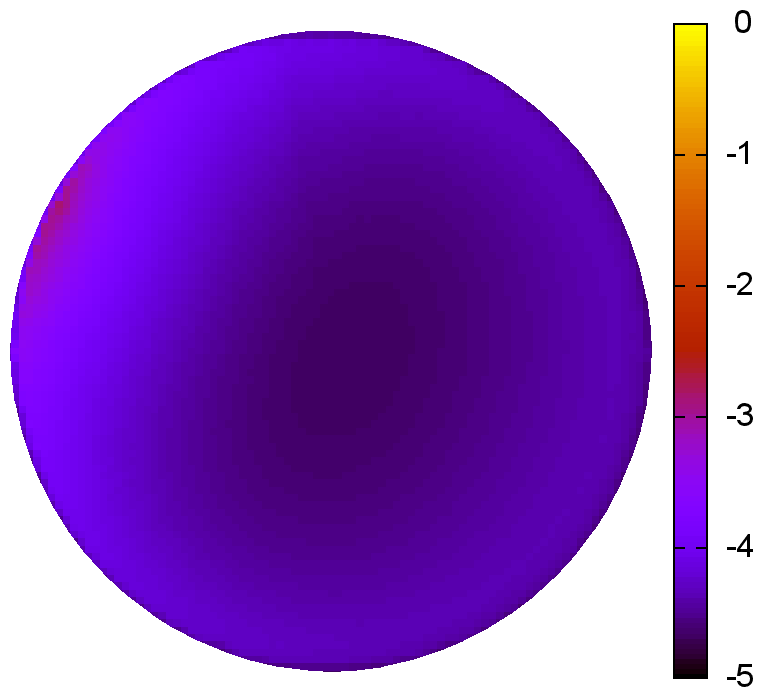 |
| (D) | 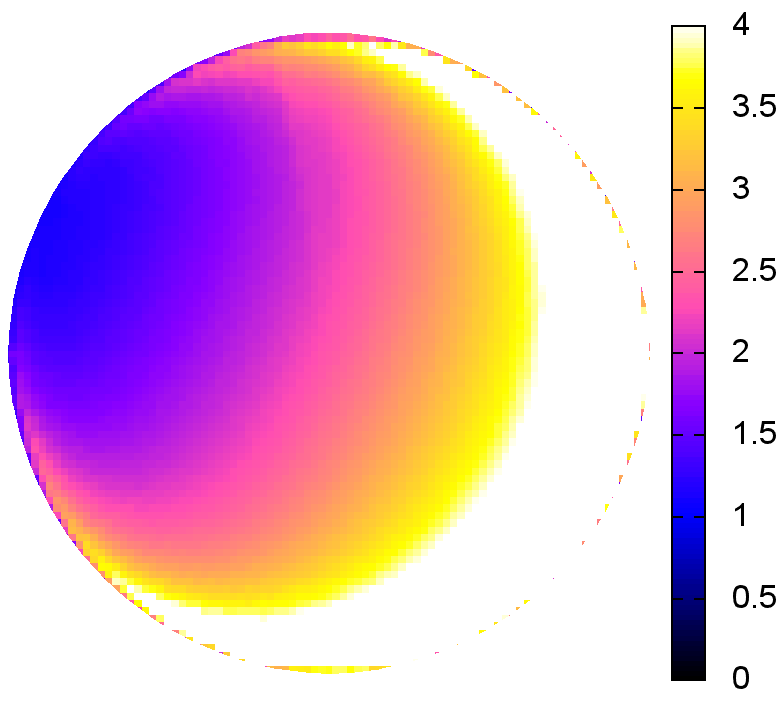 | 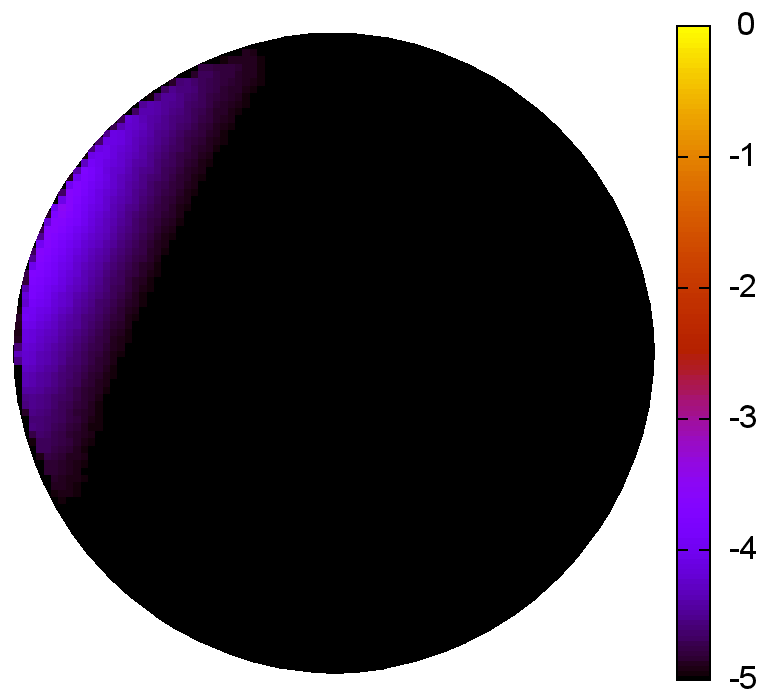 | 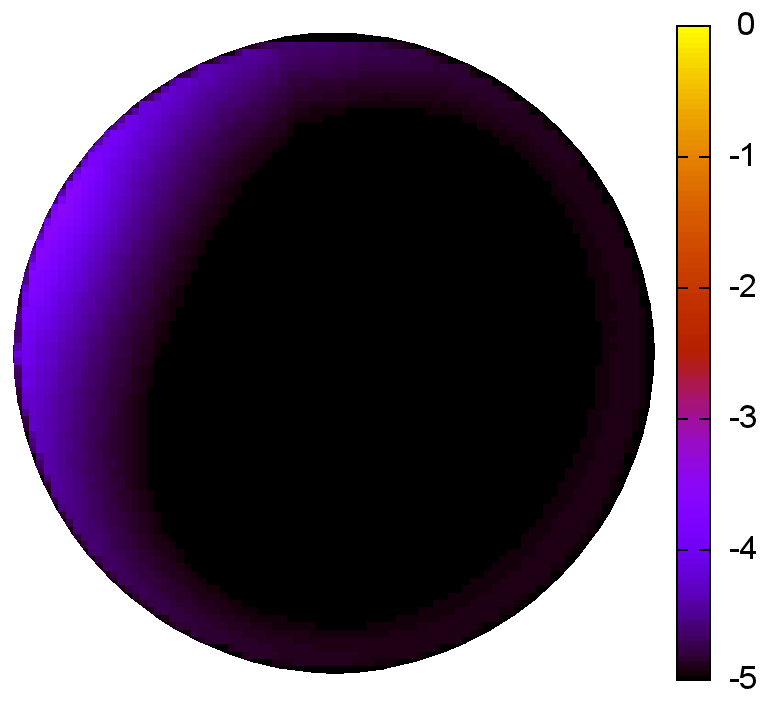 |

**Fig. S2:** **Aerosol-induced changes to NSB at a set of discrete distances, *d*, from a light source for λ = 450 nm.** The plots are organized the same way as in Fig. 2 of the main text. We also keep the same scale to make comparisons with Fig. 2 and Extended Data Fig. S3 possible.

| (A) | 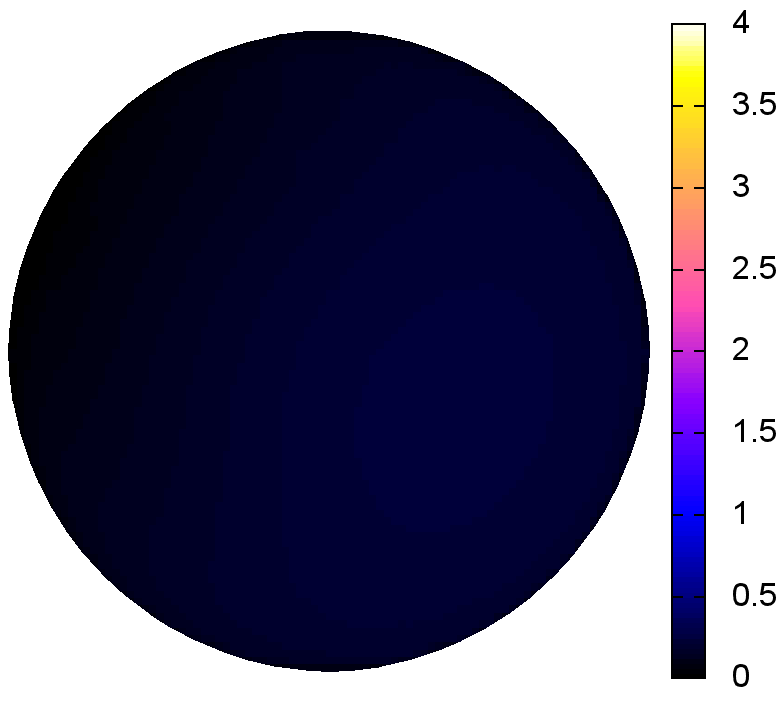 | 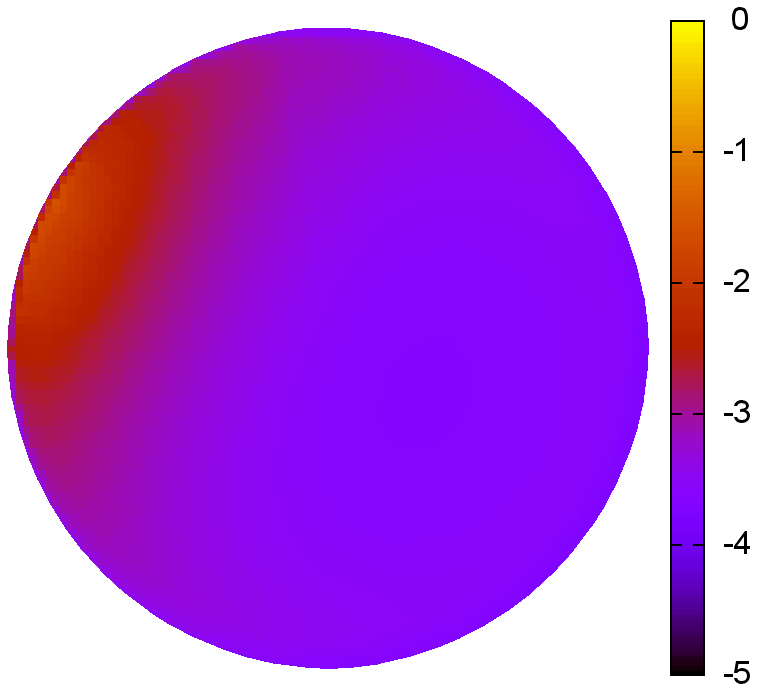 | 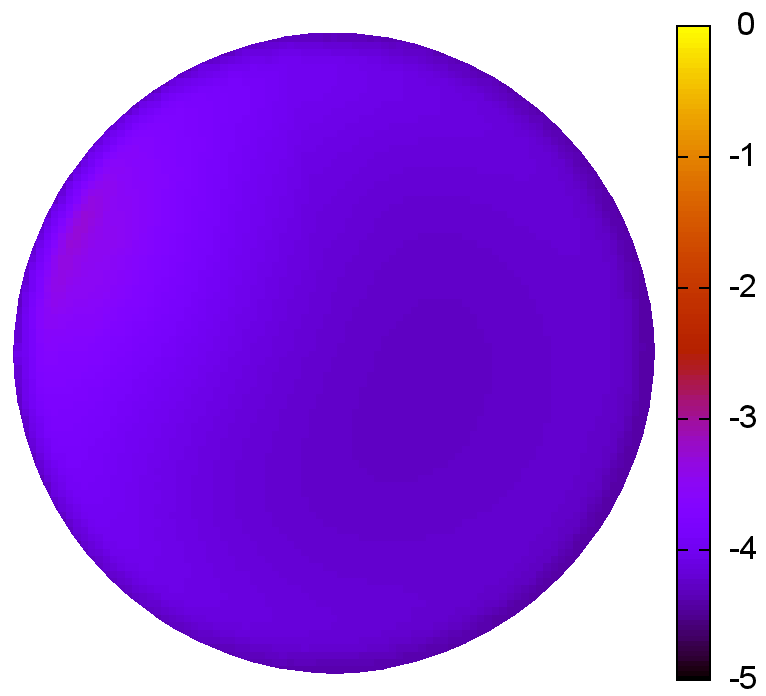 |
| --- | --- | --- | --- |
| (B) | 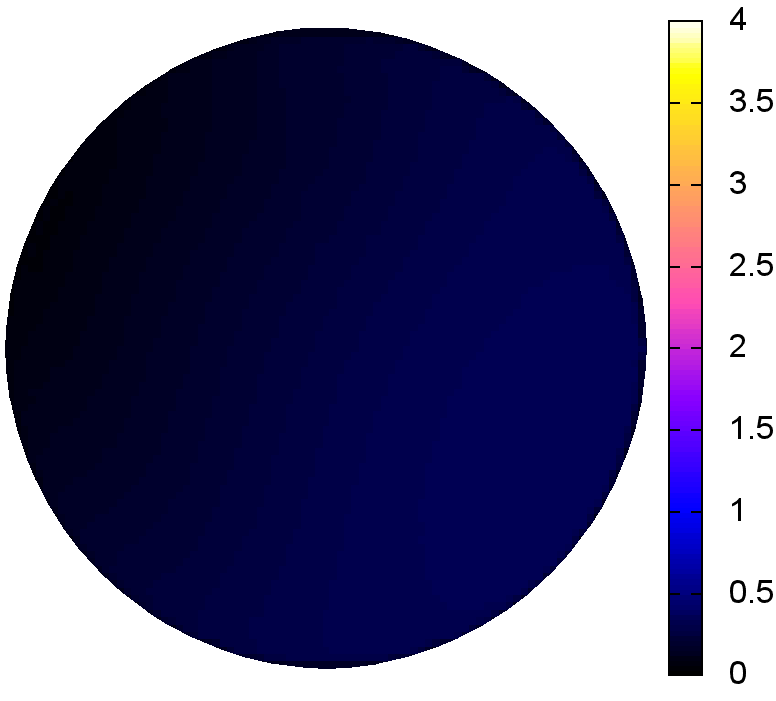 | 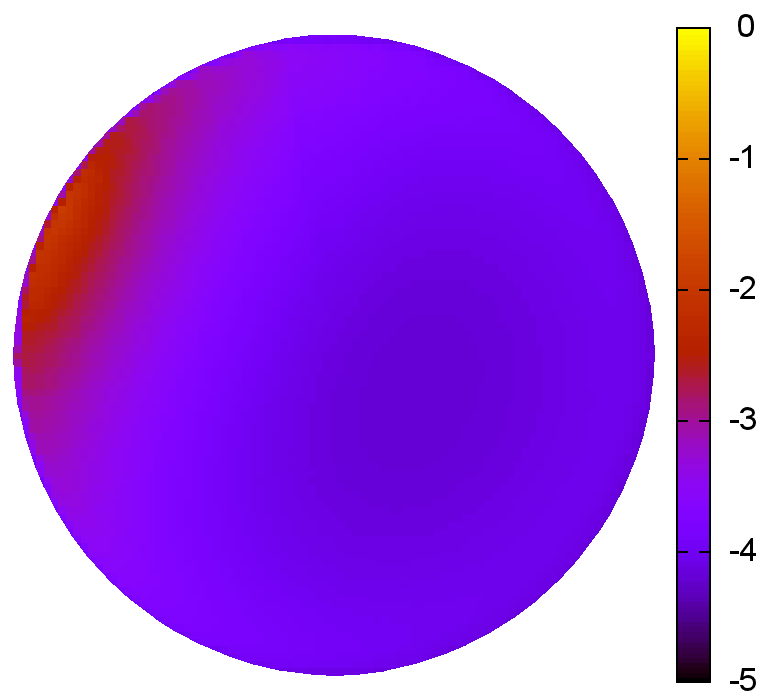 | 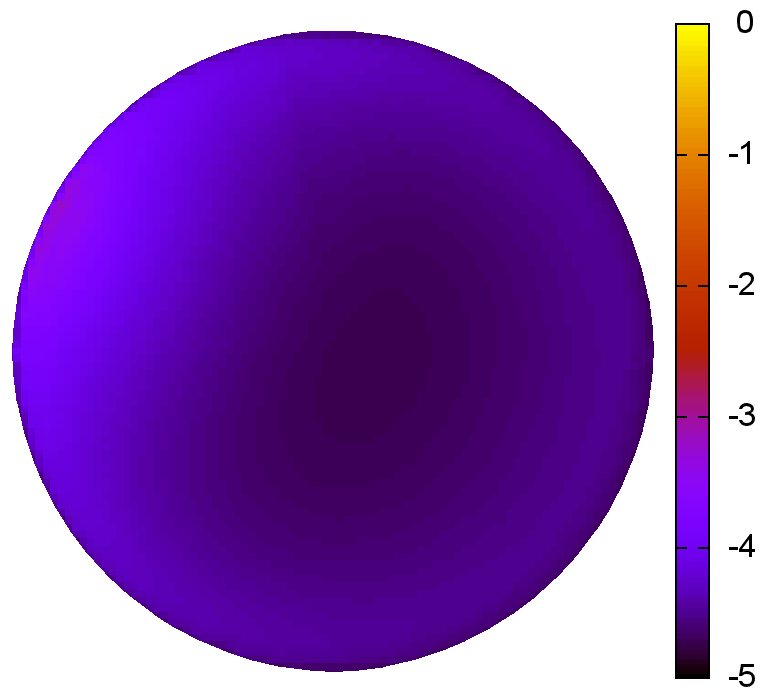 |
| (C) | 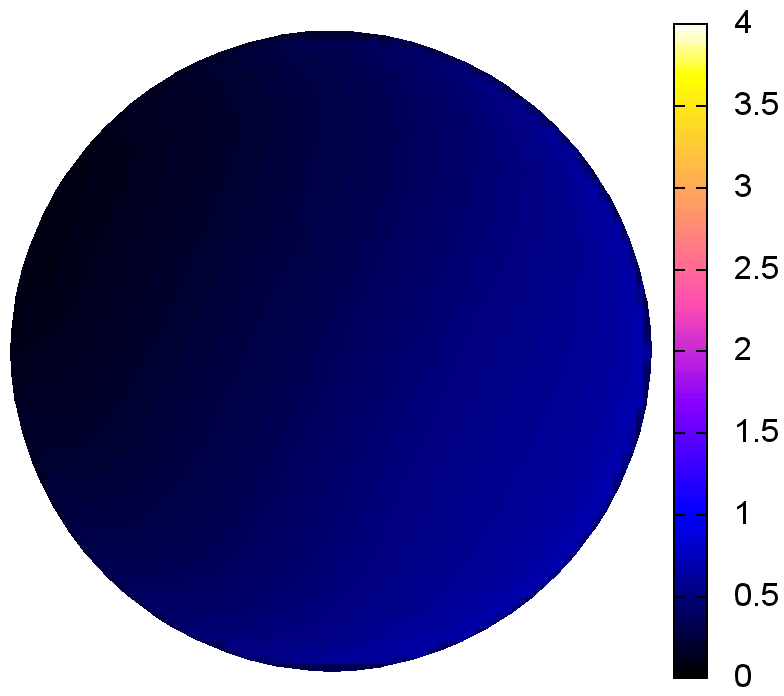 | 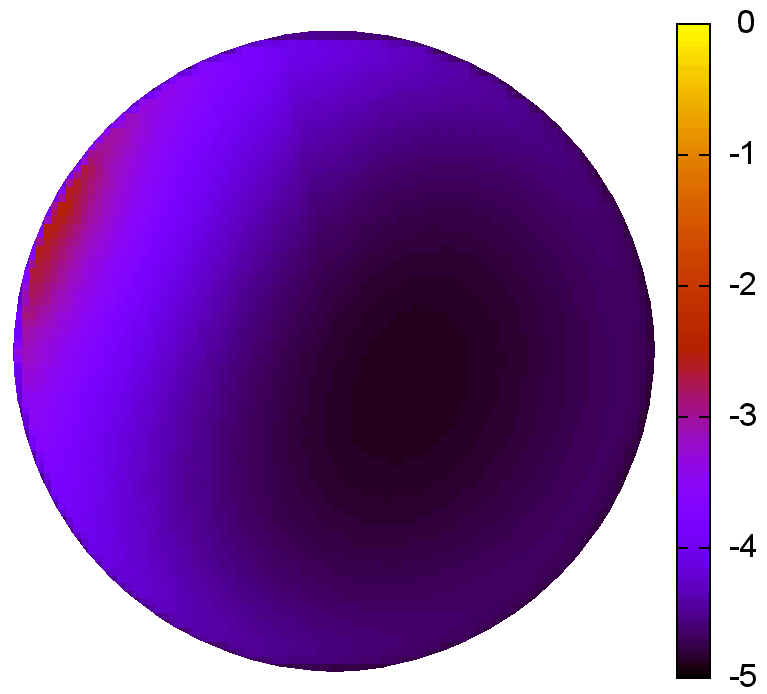 | 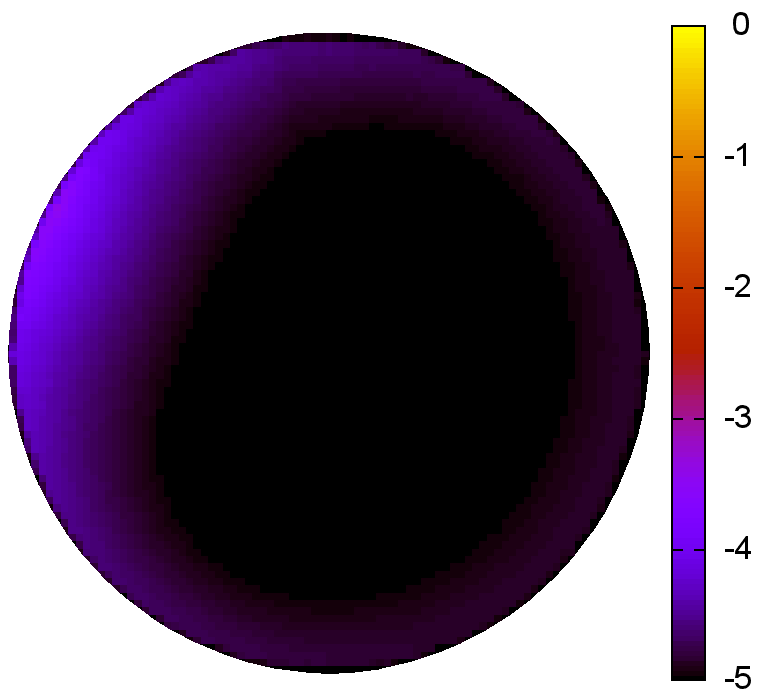 |
| (D) | 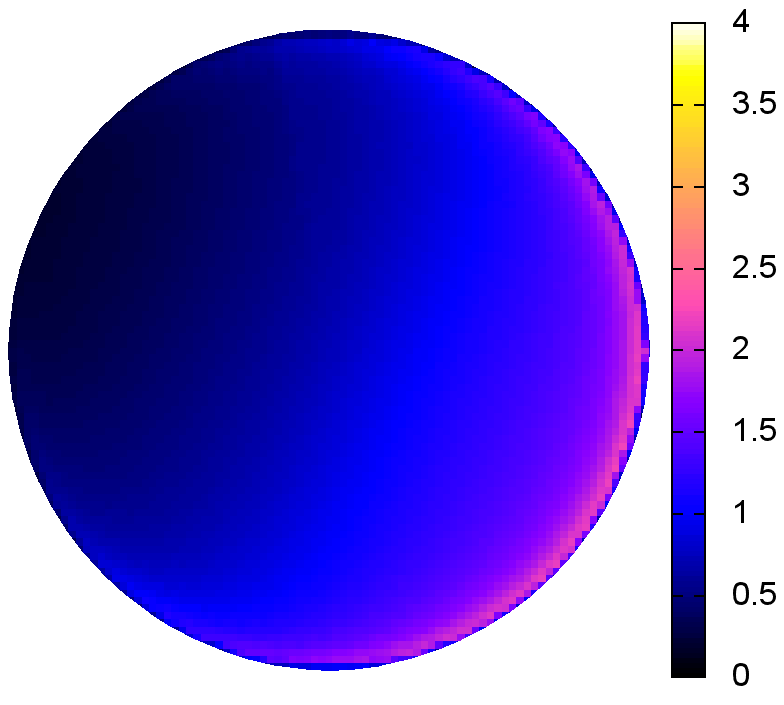 | 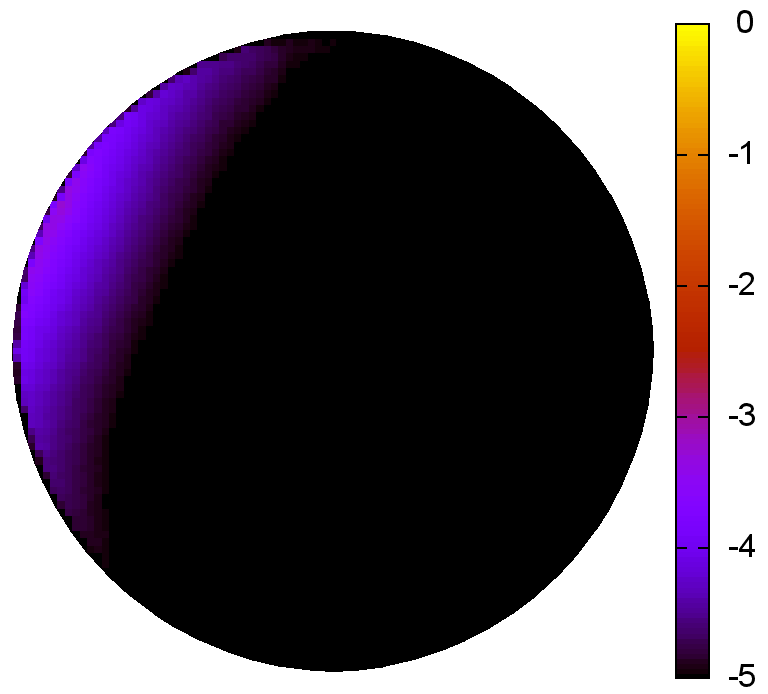 | 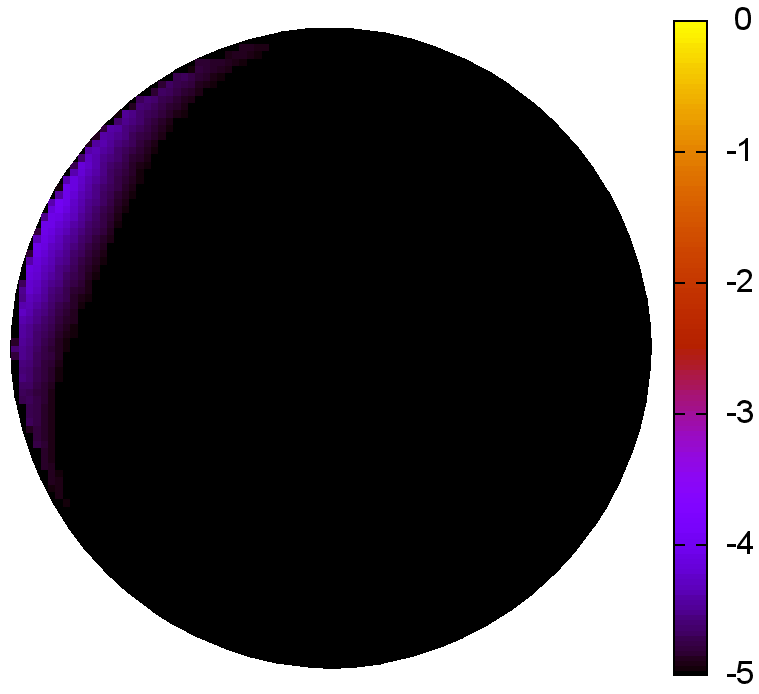 |

**Fig. S3: The same as in Fig. 2 or Fig. S2 but for λ = 650 nm.** The difference between NSB distributions in turbid atmosphere (middle pane) and unturbid atmosphere (right pane) are obvious.
